# Key Advances in Intensive Care and the Coronavirus Disease-19 Research and Practice Boost

**DOI:** 10.3390/jcm11123370

**Published:** 2022-06-12

**Authors:** Spyros D. Mentzelopoulos, George Adamos

**Affiliations:** First Department of Intensive Care Medicine, National and Kapodistrian University of Athens Medical School, Evaggelsimos General Hospital, 45-47 Ipsilandou Street, GR-10675 Athens, Greece; george.adamos1983@gmail.com

Components of intensive care include resuscitation, cardiorespiratory stabilization, reversal of organ/system dysfunction or failure, treatment of the underlying pathology, weaning from external support of vital organs, and supportive interventions (e.g., physiotherapy, psychological interventions) aimed at paving the way to an uneventful recovery and rehabilitation. Depending on patient values, goals and preferences, the holistic intensive treatment(s) may be limited or withdrawn and replaced/followed by end-of-life care interventions for the prevention or alleviation of any distressing symptoms (e.g., dyspnea, pain etc.) [1].

Current treatment recommendations for specific subgroups of critically ill patients are based on a systematic and rigorous evaluation of published evidence, including the results of randomized controlled trials (RCTs). When the Grading of Recommendations, Assessment, Development and Evaluation approach is adopted, evidence quality is rated as high, moderate, low, or very low and evidence profiles (summaries) are generated using the online Guideline Development Tool (https://gdt.guidelinedevelopment.org, accessed on 30 May 2022) [2,3,4].

Over the past decade, and especially over the past 3 years of the coronavirus disease 2019 (COVID-19) pandemic, several potentially beneficial interventions were tested in multicenter RCTs. Relevant published evidence has already been partly systematically reviewed and/or meta-analyzed. Pertinent, prominent examples include (1) noninvasive techniques of respiratory support (e.g., high-flow nasal canula, continuous positive airway pressure), prone positioning (for ≥16 consecutive hours per day with lung-protective ventilation) and veno-venous extracorporeal membrane oxygenation (ECMO) in acute respiratory distress syndrome (ARDS) of varying severity [5,6,7,8,9,10]; (2) use of RCT evidence-supported physiological targets such as ventilator driving pressure of <15 cmH_2_O during low-tidal volume ventilation in ARDS [11]; (3) adjunctive hydrocortisone with or without fludrocortisone in septic shock, and dexamethasone in ARDS (of COVID-19 or non-COVID-19 etiology) [12,13,14,15,16]; (4) targeted temperature management (e.g., hypothermia or normothermia with target temperature of 33 or ≤37.5 °C, respectively) after cardiac arrest [17,18,19]; (5) vasopressin, stress-dose steroids, and epinephrine in in-hospital cardiac arrest [20,21,22,23,24]; (6) early inhibition of fibrinolysis by tranexamic acid in acute severe bleeding due to trauma and in postpartum hemorrhage [25,26,27]; (7) nucleotide inhibition of severe acute respiratory syndrome coronavirus 2 RNA-dependent RNA polymerase [28,29]; and (8) immunomodulating interventions such as interleukin (IL)-6 receptor blockade, Janus kinase inhibition, or IL-1 alpha and IL-1 beta antagonism guided by soluble urokinase plasminogen receptor plasma levels in COVID-19 [30,31,32,33,34].

Beneficial interventions are frequently based on robust physiological, mechanistic data. For example, prior studies have shown that prone position reduces transpulmonary pressure (i.e., lung parenchymal stress) and the tidal volume to end-expiratory lung volume ratio (i.e., lung strain or tidal parenchymal deformation) in severe ARDS [35,36]. In contrast to the supine or semirecumbent position, shape matching of the ″cone-like″ lung to the “cylinder-like” chest wall and gravitational forces act in opposite directions in the prone position [37]. This attenuates the derecruitment of the dependent ventral lung units, while dorsal and medial lung units are being recruited following the relief of the supine position-associated, external compression of small airways by the abdominal contents and heart, respectively [37,38]. Supine position’s transpulmonary pressure gradient is reduced by pronation [37,38]. Whenever dorsal lung recruitment prevails over ventral lung derecruitment, pronation is associated with a lower lung stress distributed more homogenously over an increased number of aerated lung units [35,37,38]. Concurrently, dorsal lung perfusion is maintained, resulting in improved ventilation-perfusion matching, reduced shunt fraction, and improved oxygenation [35,37,38]. Carbon dioxide clearance may also improve following pronation, partly because of reduced overdistention of the dependent, ventral lung, and concurrent sparing from overdistention of the nondependent, dorsal lung [37]. Pronation may result in reduced dead space ventilation and lower PaCO_2_ [35], and these physiological benefits may translate into improved survival to hospital discharge [39].

During the COVID-19 pandemic, intensive care practice was guided by the prompt issuance of guidelines including recommendations based on both direct and indirect (i.e., extrapolated from other viral pneumonias) evidence [4] and by an abundance of concurrently emerging RCT data [8,28,29,30,31,32,33,34]. Furthermore, two simplified models of COVID-19-related ARDS (CARDS) were proposed as opposite extremes of a pathophysiological spectrum that includes “intermediate stages” with overlapping characteristics. The least severe form of CARDS (termed “type L”) comprises low lung elastance and weight, and is relatively unresponsive to positive end-expiratory pressure (PEEP). The most severe form (termed “type H”) comprises extensive computerized tomographic consolidations, high lung elastance and weight, and is responsive to PEEP [40]. In this context, it was postulated that high lung stress secondary to vigorous, spontaneous inspiratory effort during “type L” CARDS may result in patient’s self-inflicted lung injury, thereby expediting transition to “type H” CARDS [40,41]. Accordingly, timely endotracheal intubation of hypoxemic/hypercapnic COVID-19 patients with evidence of high breathing work (e.g., phasic contraction on palpation of the sternomastoid muscle) has been suggested [41,42].

The COVID-19 mass casualty crisis and dismal outcomes of severe CARDS have also prompted the introduction and/or preliminary evaluation of interventions such as awake prone positioning and pronation during ECMO, respectively. Recent physiological data suggest that awake pronation may reduce the respiratory rate and work of breathing in CARDS patients supported by continuous positive airway pressure [43]. However, in a recent RCT of 400 CARDS patients receiving noninvasive respiratory support, awake pronation did not significantly reduce intubation rates or in-hospital mortality, and this mandates further evaluation in larger RCTs [44]. Pronation might also disrupt a potentially vicious cycle of ongoing native lung damage during ECMO [45]. In a recent meta-analysis, pronation during ECMO improved oxygenation, reduced driving pressure, and was associated with a cumulative survival rate of 57%; however, it was also associated with prolonged ECMO runs and ICU length of stay [46].

The COVID-19-associated, compelling need for new and effective life-sustaining and curative interventions in the presence of periodic healthcare systems’ saturation has also prompted the issuance of ethical guidelines including evidence-based recommendations about advance care planning, shared decision making, and rationing of resources [47,48]. Ethical, legal, and pandemic-related challenges pertaining to ECMO use in cardiac arrest have also been analyzed [49].

The current special issue on “Key Advances in the Treatment of the Critically Ill” primarily aims to highlight major aspects of the rapidly evolving knowledge of the mechanisms and pathophysiology of critical illness (including COVID-19), and the rapidly accumulating evidence on the efficacy of new life-sustaining and/or therapeutic interventions. Reports on the ethics of end-of-life decisions and practices are also encouraged.
